# Comparison of Adult Testis and Ovary MicroRNA Expression Profiles in Reeves’ Pond Turtles (*Mauremys reevesii*) With Temperature-Dependent Sex Determination

**DOI:** 10.3389/fgene.2020.00133

**Published:** 2020-03-02

**Authors:** Lei Xiong, Mengli Yang, Kai Zheng, Ziming Wang, Shengli Gu, Jiucui Tong, Jianjun Liu, Nadar Ali Shah, Liuwang Nie

**Affiliations:** ^1^ Life Science College, Provincial Key Lab of the Conservation and Exploitation Research of Biological Resources in Anhui, Anhui Normal University, Wuhu, China; ^2^ Biochemistry Department, Wannan Medical College, Wuhu, China

**Keywords:** *Mauremys reevesii*, microRNA, ovary, testis, temperature-dependent sex determination (TSD)

## Abstract

Some differentially expressed genes (DEGs) that encode key enzymes involved in steroidogenic biosynthesis (*CYP19A1*) and key molecules related to gonadal functions (*DMRT1*, *SOX9*, *AMH*, *FOXL2*, *WNT4*, *RSPO2*, and *GDF9*) have been identified in adult gonadal RNA-seq studies of Reeves’ pond turtle (*Mauremys reevesii*) with temperature-dependent sex determination (TSD). Gonadal functional maintenance and gametogenesis comprises a highly regulated and coordinated biological process, and increasing evidence indicates that microRNAs (miRNAs) may be involved in this dynamic program. However, it is not clear how the regulatory network comprising miRNAs changes the expression levels of these genes. In this study, miRNA sequencing of adult testis and ovary tissues from *M. reevesii* detected 25 known and 379 novel miRNAs, where 60 miRNAs were differentially expressed in the testis and ovary. A total of 1,477 target genes based on the differentially expressed miRNAs were predicted, where 221 target genes also exhibited differential expression. To verify the accuracy of the sequencing data, 10 differentially expressed miRNAs were validated by quantitative reverse transcription real-time PCR, and were found to be consistent with the transcriptome sequencing results. Moreover, several miRNA/target gene pairs, i.e., mre-let-7a-5p/mre-let-7e-5p and *CYP19A1*, mre-miR-200a-3p and *DMRT1*, mre-miR-101-3p and *SOX9*, and mre-miR-138-5p and *AMH* were identified. To explore the regulatory role of miRNAs, we conducted target gene enrichment analysis of the miRNAs and 221 target genes in the regulatory network. The signaling pathways related to gonadal functional maintenance and gametogenesis based on the DEGs and target genes were then compared. Our findings provide crucial information to facilitate further research into the regulatory mechanisms involving miRNAs in turtle species with TSD.

## Introduction

Both adult testes and ovaries play central roles in the reproductive system by secreting steroid hormones and producing gametes (Hossain et al., 2012). Gonadal functional maintenance and gametogenesis depend on the normal development of both the gonadal somatic cells and germ cells. A complex and coordinated molecular program orchestrates the correct differentiation of reproductive cell types and the maintenance of their functions in adulthood. In mammals with significant sex chromosomal differentiation, the Y chromosome gene SRY (sex-determining region Y) directs somatic cells to Sertoli cells and germ cells toward spermatogenesis (Mäkelä et al., 2018). However, species with temperature-dependent sex determination (TSD) have almost identical genetic material in males and females, and their gonads have functional sex reversal potential, but adult TSD turtles do not undergo sex reversal spontaneously, and the regulatory mechanism in maintaining the phenotype is still unclear. Gonadal functional maintenance and gametogenesis comprises a highly regulated and coordinated biological process. Many sex-related genes that have been identified in mammals and birds also exist in TSD turtles, and previous studies have found significant differences in gene expression in the adult gonads of the Reeves’ pond turtle (*Mauremys reevesii*) (Xiong et al., 2019). Increasing evidence indicates that microRNAs (miRNAs) may be involved in this dynamic process (Dai and Ahmed, 2014; Wang et al., 2016). However, it is not clear how the regulatory network comprising miRNAs changes the expression levels of these genes.

Several studies have explored the critical roles of miRNAs in testis development and spermatogenesis (Kotaja, 2014; Pratt and Calcatera, 2016), such as let-7 miRNA, which can promote the differentiation of germ cell fates in *Caenorhabditis elegans* (Reinhart et al., 2000), as well as oocyte growth, maturation, and development during the regulation of oogenesis (Juanchich et al., 2013), e.g., miR-101 regulates ovary differentiation in chicken gonads (Kang et al., 2013). In TSD turtles, the regulatory function of miRNAs was first examined in the red-eared slider turtle (*Trachemys scripta*) (Biggar and Storey, 2012). At present, 405 miRNAs have been identified in miRBase 22.0 (http://www.mirbase.org/) for only one turtle species, *Chrysemys picta* (Biggar and Storey, 2015), which suggests that more miRNAs await characterization in turtles. Next generation sequencing facilitates the profiling of both known and novel miRNAs, especially those expressed in low abundance (Liu et al., 2018).

The freshwater turtle *M. reevesii*, belongs to the family Geoemydidae and is widely distributed in east Asia. It is a classic TSD species, and there is a male bias at low temperatures and a female bias at high temperatures (Ru et al., 2017). In our previous transcriptomic analysis of adult gonads in *M. reevesii*, we identified 1,594 differentially expressed genes (DEGs) and demonstrated the differential expression of genes involved in four signaling pathways related to hormone synthesis and gametogenesis (Xiong et al., 2019). Given the large number of DEGs that regulate these pathways, miRNAs may have roles as regulators of DEGs. Therefore, in this study, we identified miRNAs in *M. reevesii* by deep sequencing to compare their tissue-specific expression levels in the testis and ovary, as well as predicting the putative target genes that mapped to these differentially expressed miRNAs. Moreover, combined with previous transcriptome studies, we compared the signaling pathways related to gonadal functional maintenance and gametogenesis for the DEGs, and conducted target gene enrichment analysis based on the miRNAs and target gene regulatory network. These findings expand the list of miRNAs annotated for turtles and provide crucial genomic information to facilitate further research into the regulatory mechanisms involving miRNAs related to gonadal maintenance and gametogenesis during sexual maturity in turtle species with TSD.

## Materials and Methods

### Ethics Statement

Procedures involving animals and their care were approved by the Animal Care and Use Committee of Anhui Normal University (#20170612).

### Tissue Collection, RNA Preparation, and Generation of Small RNA Sequencing Libraries

Six adult turtles (three males, three females) aged 6 years were obtained from Wuhu (31°33′N, 118°370′E, southeast China) in 2017. Gonad samples (testicles and ovaries) were collected and dissected (**Figure S1**). The gonad samples were flash frozen in liquid nitrogen and stored at −80 °C until the RNA was extracted (Yin et al., 2016).

RNA was extracted from each sample with TRIzol reagent (Invitrogen, Carlsbad, CA, USA) according to the manufacturer’s instructions. Six small RNA libraries were constructed using an Illumina TruSeqTM Small RNA Sample Preparation kit (Illumina, San Diego, CA, USA). Sequencing was performed using the Illumina Hiseq 2500 platform by Genergy Bio-technology Co. Ltd (Shanghai, China). The raw data are available in the NCBI Sequence Read Archive (GenBank accession no. PRJNA542219).

### Sequencing Data Processing

The raw small RNA reads were processed with FastQC v0.11.5 (https://www.bioinformatics.babraham.ac.uk/projects/fastqc/) using the PHRED algorithm and the low-quality reads and adaptor contamination were removed using cutadapt v2.4 (Martin, 2011), thereby resulting in fragments corresponding to RNAs with lengths of 15–40 nucleotides (nt). Subsequently, small RNA reads were aligned against expressed sequence tags (ESTs) stored in NCBI (https://blast.ncbi.nlm.nih.gov), Rfam 11.0 (Burge et al., 2012), and RepBase (Jurka et al., 2005). Filtering was performed by removing reads that mapped to rRNA, tRNA, small nuclear RNA (snRNA), and small nucleolus RNA using bowtie v1.2.1.1 (Langmead et al., 2009).

### miRNA Identification and Differential Expression Analysis

We used miRDeep2 software v2.0.0.8 (Friedländer et al., 2008) for miRNA identification based on the clean sRNA reads. Putative novel miRNAs predicted by miRDeep2 were retained for further analysis if they had a score ≥5, which corresponded to an estimated false discovery rate of 6%. The Mfold program based on free energy minimization (Zuker, 2003) was used to predict their propensity to form hairpin loops as potential pre-miRNAs. Sequences of mature miRNAs from all animal species were downloaded from miRBase 22.0 and combined to obtain all known animal miRNA sequences. The known miRNAs in *M. reevesii* were identified by BLAST search against all known animal miRNA sequences with no mismatch. A Venn diagram based on *M. reevesii* and *C. picta*, *Anolis carolinensis*, and *Mus musculus* mature miRNAs sequences was prepared. To quantify miRNA expression, tags per million reads (TPM) was used to normalize the miRNA expression levels. Differentially expressed miRNAs were determined using the R package edgeR (Robinson et al., 2010) where each tissue was analyzed individually. The statistical *P*-values were adjusted using Benjamini and Hochberg’s (1995) approach to control the false discovery rate. The differentially expressed miRNAs were identified according to the following criteria: (1) |log2(FC)| > 1; and (2) a corrected *P*-value <0.05.

### miRNA Target Predictions and Construction of miRNA Target Network

MiRanda tools (Enright et al., 2003) and Targetscan v3.1 (Lewis et al., 2005) were used to predict the potential targets of the differentially expressed miRNAs (Jiang et al., 2018). TargetScan (Riffo-Campos et al., 2016) was used to detect whether the 3′-untranslated region (3′UTR) of the mRNA in each pair matched the seed region of miRNA. Only when the target was identified by both programs, was it considered to be the potential target for a given miRNA. Finally, the data predicted by miRanda and TargetScan v3.1 were combined and the overlaps were calculated. Then miRNA–mRNA interactions were calculated according to miRNA expression profiles and transcriptome data, negatively correlated miRNA–mRNA pairs were determined using Pearson correlation analysis, and miRNA–gene networks were produced and visualized using Cytoscape v3.7.1 software (Shannon et al., 2003). The basic functional targets were classified using Gene Ontology (GO) (http://www.geneontology.org/) annotations based on Blast2GO v2.5 with an *e*-value of 1*e*−6 (Young et al., 2010) and the Kyoto Encyclopedia of Genes and Genomes (KEGG) pathway database (http://www.genome.jp/kegg/) based on KAAS with an *e*-value of 1*e*−10 (Mao et al., 2005), where a *P*-value <0.05 was defined as statistically significant. These analyses were based on human annotation using the Database for Annotation, Visualization, and Integrated Discovery (DAVID) web server (http://david.abcc.ncifcrf.gov/) with the EASE value set to 0.05 (Huang et al., 2007). Visualizations of the analyses, including GO and KEGG enrichment results, were performed in the R (v3.4.2) package with ggplot2.

### Quantitative Real-Time PCR (qRT-PCR) Validation of Differentially Expressed miRNAs in *M. reevesii*

To verify the relationships between mRNAs and miRNAs, we selected genes with significant differences in expression in the steroid hormone biosynthesis pathway based on KEGG pathway analyses, where the differentially expressed miRNAs were identified as candidate regulators of these genes. Finally, 10 miRNAs were selected to validate their expression profiles by qRT-PCR. All of the reactions were performed using three technical replicates and three biological replicates to validate the reliability of the predicted miRNAs. The expression levels of miRNAs were determined with the ABI StepOnePlus system (Applied Biosystems, Foster, CA, USA) using qRT-PCR reagents provided by Toyobo and Beacon Real-Time PCR Universal Reagent (Cat#GMRS-001, GenePharma, Shanghai). The specific RT-PCR primers and stem-loop primers used for miRNA quantification are shown in **Table S1**. U6 snRNA was used as an internal control. The thermal cycling program comprised denaturation at 95°C for 3 min, followed by 40 cycles of amplification with denaturation at 95°C for 12 s, annealing at 62°C for 30 s, and extension at 72°C for 30 s. Melting curve analysis was conducted from 60 to 95°C. The expression of each miRNA relative to U6 was calculated using the 2^−ΔΔCT^ method, as described previously (Livak and Schmittgen, 2001). Statistical analyses of the qRT-PCR results were carried out in GraphPad Prism software v6.0 (San, Diego, CA, USA). Statistical significance of the data was tested by performing paired t-tests. The results are presented as means ± SEM of three replicates, and the statistical significance is represented by *P*-value <0.05.

## Results

### Profiling of Sequencing Data

To identify the miRNAs in *M. reevesii*, six small RNA libraries were constructed and sequenced using Illumina HiSeq 2500. In total, 11,965,294; 19,268,984; 8,852,158; 10,989,442; 12,196,601, and 17,646,067 raw reads were obtained, respectively, in the small RNA libraries. These reads were first adjusted to remove any sequencing artifacts, including reads without a 3′ adaptor, reads measuring <15 nt and >40 nt, and junk reads. After data processing, 10,143,882; 15,910,350; 7,812,047; 9,657,812; 11,058,767; 16,836,764 clean reads were obtained from the total reads in the six small RNA libraries (**Table S2**).

### Prediction of Potential miRNAs

**Figure 1:**
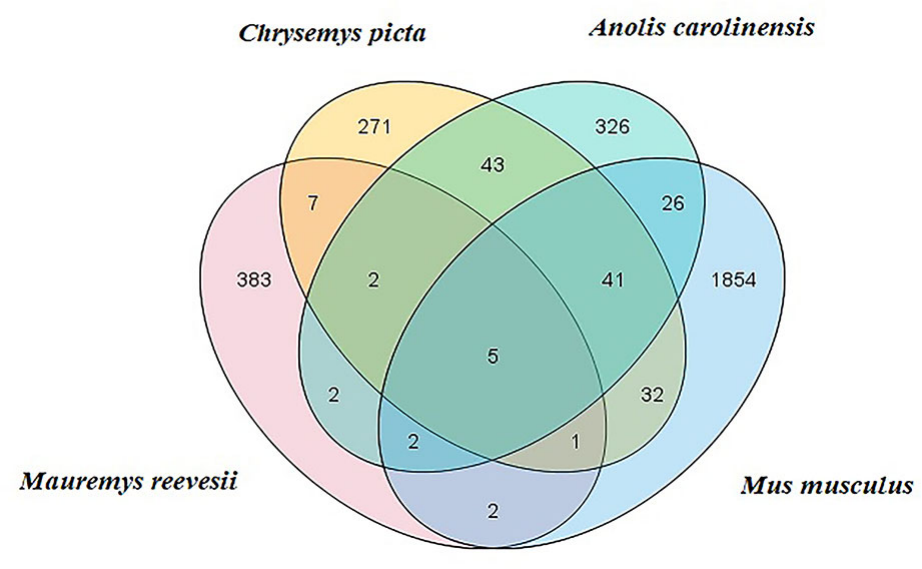
*M. reevesii* miRNAs compared with *Chrysemys picta*, *Anolis carolinensis*, and *Mus musculus* in miRBase v22. The intersection shows the number of conserved miRNAs.

Clean reads were mapped to Rfam, RepBase, EST database, and miRBase (v22). The size distribution of the clean reads is shown in **Figure S2**. The majority of the clean reads measured 20–24 nt in length. In total, 3,369 unique reads were mapped to the *C. picta bellii* genome using miRDeep2 (v2.0.0.8) and miRBase v22. The unique reads from the six small RNA libraries were aligned with all known animal miRNA sequences using BLASTN search. We identified some putative novel miRNAs using reads that did not map to known miRNAs. In total, 404 miRNAs were identified comprising 25 known (**Table S3**) and 379 novel miRNAs (**Table S4**), which were classified into 23 miRNA families. Based on comparisons with the *C. picta*, *A. carolinensis*, and *M. musculus* databases in miRBase v22, five miRNAs were found to be co-expressed in the four species, seven miRNAs were found in *C. picta* and *A. carolinensis*, six miRNAs were found in *C. picta* and *M. musculus*, and seven miRNAs were found in *M. musculus* and *A. carolinensis* according to Venn diagrams (**Figure 1**).

### miRNA Differential Expression Profiles

We then compared the transcription levels of the differentially expressed miRNAs in the ovary and testis. After strict filtration (corrected *P*-value <0.05, |log2(FC)| > 1), 43 miRNAs were significantly upregulated in the testis, such as mre-let-7e-5p, mre-let-7a-5p, mre-miR-133c-3p, and mre-miR-16a-5p, whereas 17 miRNAs were significantly upregulated in the ovaries, such as mre-miR-138-5p, mre-miR-101-3p, mre-miR-30c-5p, mre-miR-128-3p, mre-miR-200a-3p, mre-miR-1b-3p, mre-miR-2188-5p, mre-miR-200a-5p, and mre-miR-34a-5p (**Table S5**). Among these differentially expressed miRNAs, mre-novel34-5p and mre-novel199-3p were only found in the ovaries and 19 miRNAs were only found in the testis, such as mre-novel238-3p.

### Prediction of Target Genes and Enrichment Analysis

**Figure 2:**
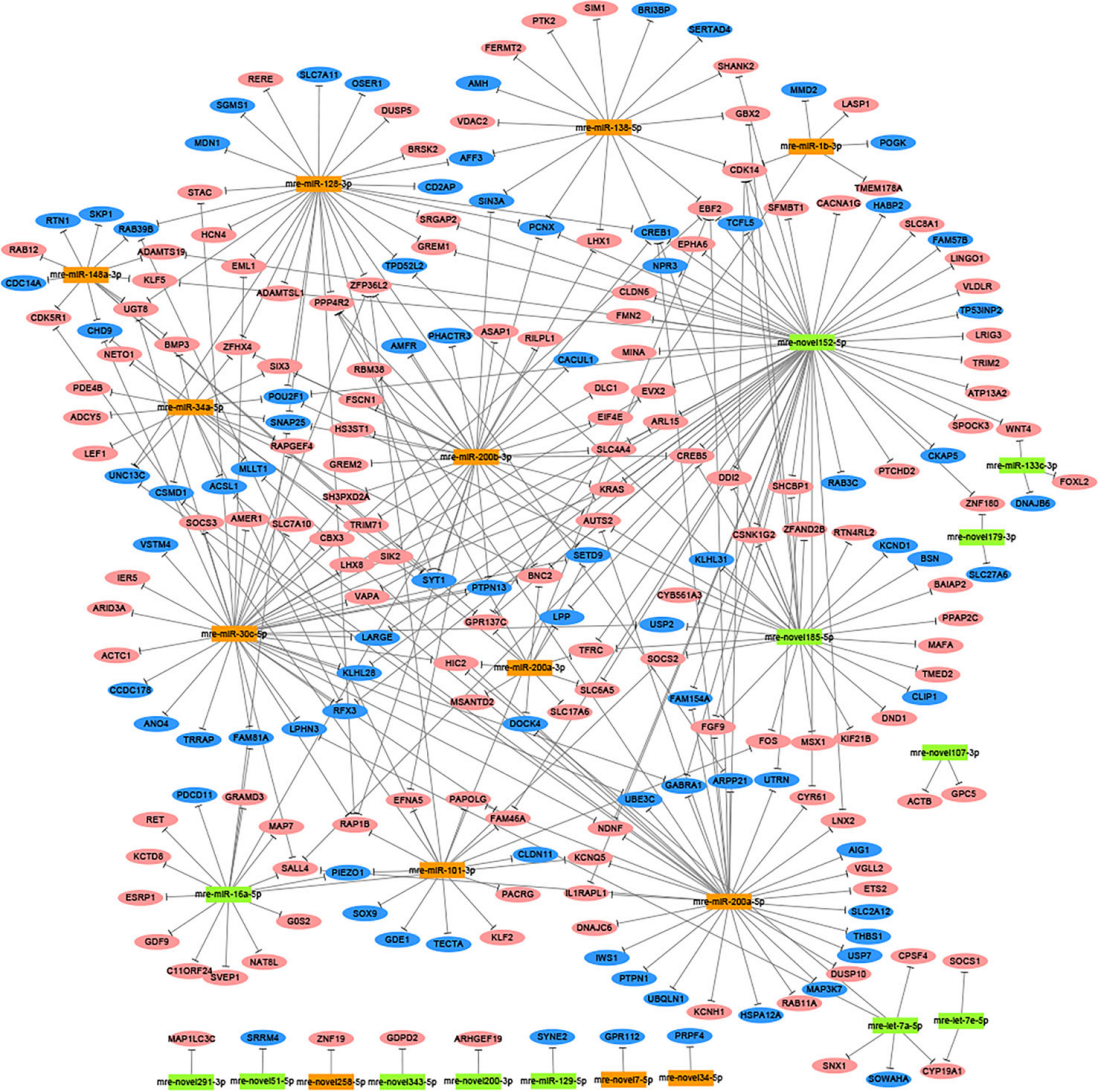
Network analysis based on the interactions among differentially expressed miRNAs and 221 potential target genes with negatively correlated expression (corrected *P*-value < 0.05). Green boxed nodes represent upregulated miRNAs in male gonads and orange boxed nodes represent upregulated miRNAs in female gonads. Pink ellipses represent downregulated genes in male gonads and blue ellipses represent downregulated genes in female gonads.

In total, 1,477 target genes were predicted for the 60 differentially expressed miRNAs using miRanda and Targetscan. These predictions suggested that a single miRNA might target more than one mRNA, such as mre-miR-30c-5p, which was predicted to target 181 genes (**Table S6**). Similarly, one gene could be controlled by one or more miRNAs, e.g., hypermethylated in cancer 2 (*HIC2*) had three miRNA target sites (mre-miR-30c-5p, mre-let-7a-5p, and mre-miR-200a-3p) (**Table S6**). In our recent study, we identified 1,594 DEGs in the adult testis and ovary using RNA-seq (Xiong et al., 2019), where 221 DEGs were also target genes predicted for the differentially expressed miRNAs, with 82 DEGs upregulated in the testis and 139 DEGs upregulated in the ovary. In these 221 DEGs, several miRNA/target mRNA pairs were identified, i.e., membrane palmitoylated protein 5 (*MPP5*) and mre-miR-34a-5p, actin beta (*ACTB*) and mre-novel107-3p, mitogen-activated protein kinase 9 (*MAPK9*) and mre-novel238-3p, dachsous cadherin-related 1 (*DCHS1*) and mre-miR-124-3p, and mitogen-activated protein kinase 8 (*MAPK8*) and mre-let-7a-5p. Although most of the predicted miRNA–target relationships need to be further validated experimentally, these results strongly suggest that miRNAs could play critical roles in regulating the functions of the ovary and testis (**Figure 2**).

**Figure 3:**
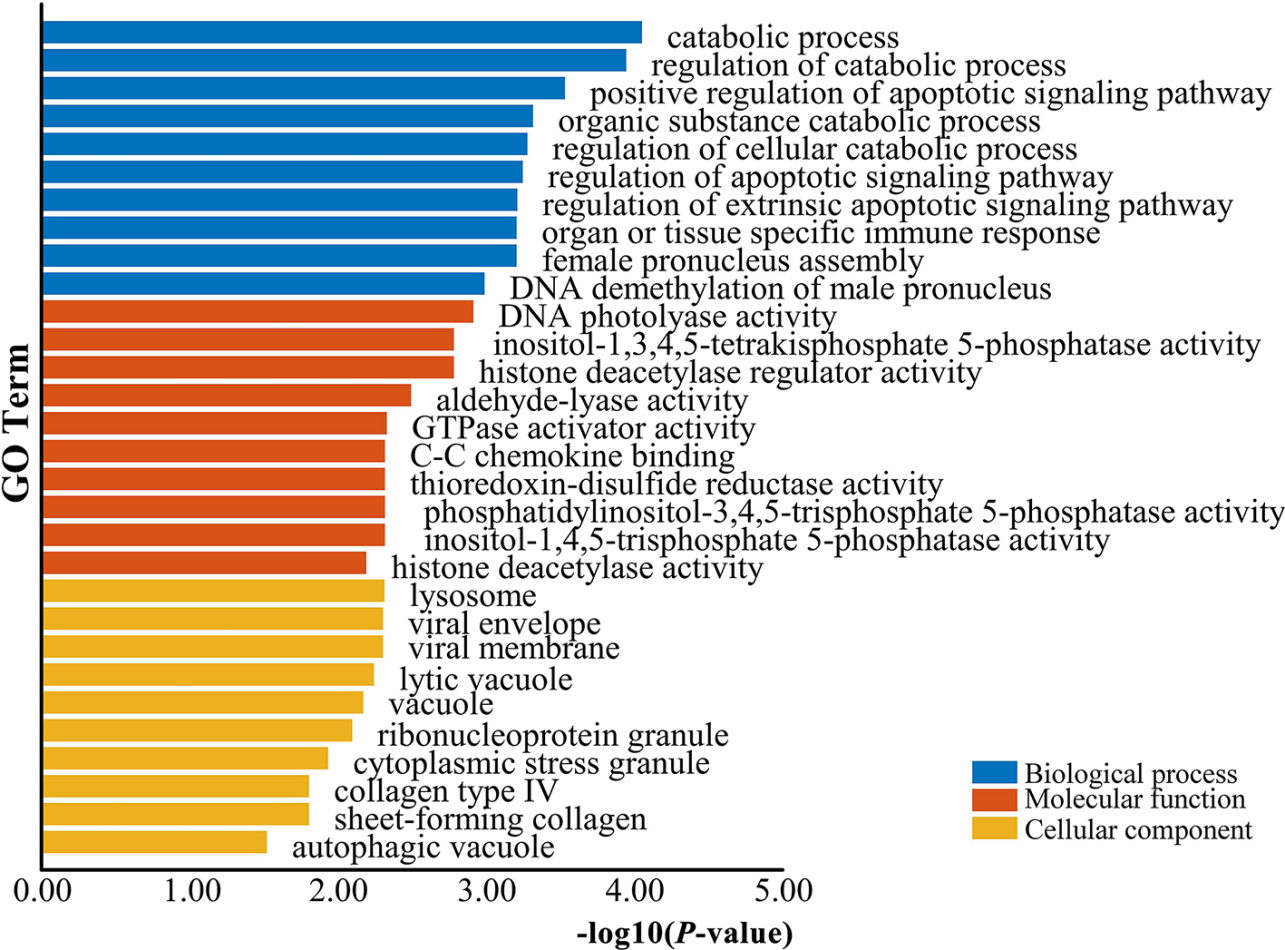
GO enrichment analysis for target genes of differentially expressed miRNAs. Blue represents the top 10 GO terms of biological process, orange represents the top 10 GO terms of molecular function, and yellow represents the top 10 GO terms of cellular component.

**Figure 4:**
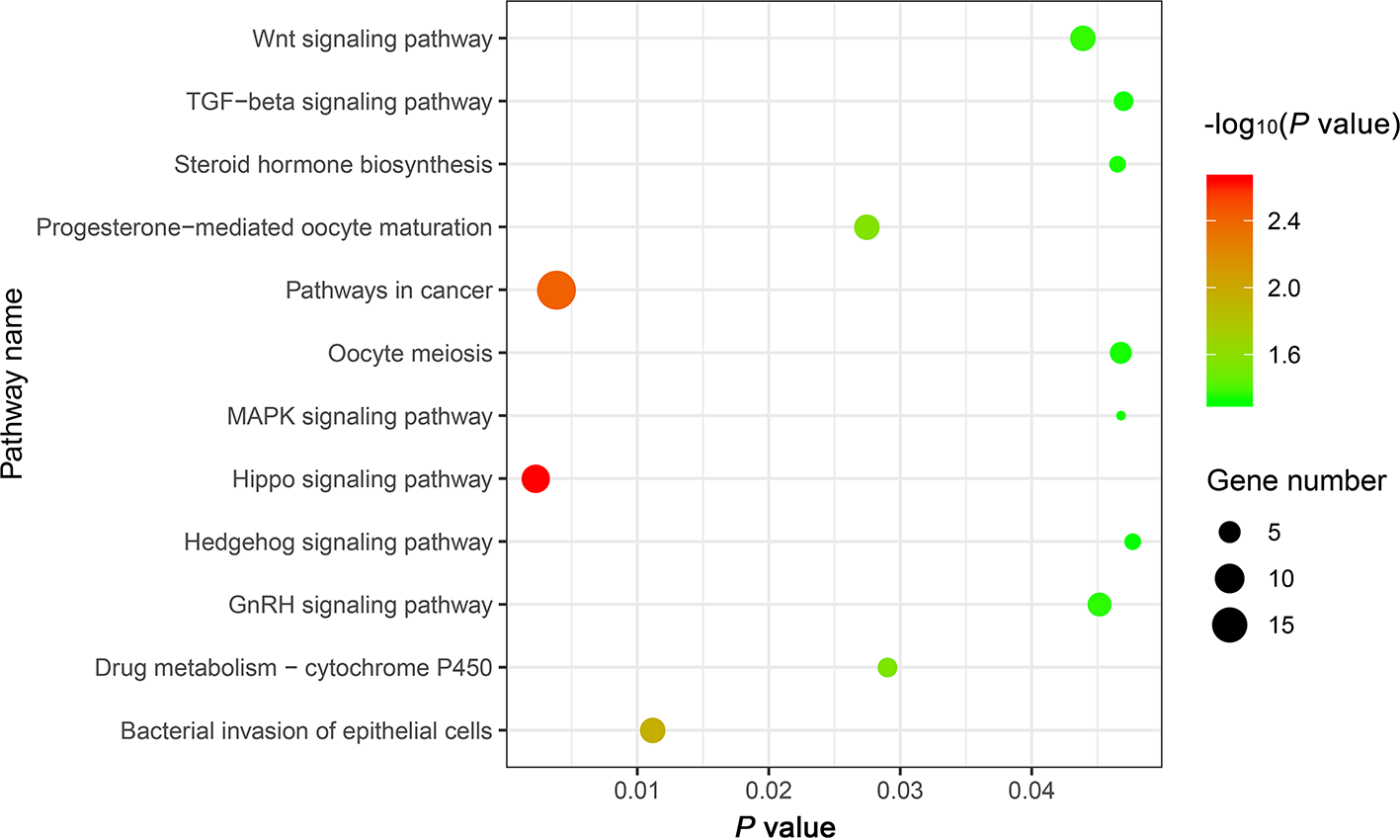
The top 12 KEGG enrichment analysis terms for target genes of differentially expressed miRNAs.

The genes potentially regulated by miRNAs according to the present study were annotated using GO annotations (**Table S7**) and KEGG pathway analyses (**Table S8**). The GO annotations were classified as cellular component, biological process, and molecular function (*P < *0.05) (**Figure 3**). We found that many of the miRNAs detected in this study are involved in catabolic processes. Based on the GO terms, we found two GO annotations related to gonadal function: female pronucleus assembly and DNA demethylation of male pronucleus. KEGG pathway analysis demonstrated that the target genes were related to significantly expressed miRNAs. According to the KEGG pathway analysis results (*P < *0.05), 12 pathways were significantly enriched, including the Hippo signaling pathway, Wnt signaling pathway, TGF-beta signaling pathway, Hedgehog signaling pathway, Steroid hormone biosynthesis, and Oocyte meiosis (**Figure 4**).

### qRT-PCR Validation of miRNA Expression

**Figure 5:**
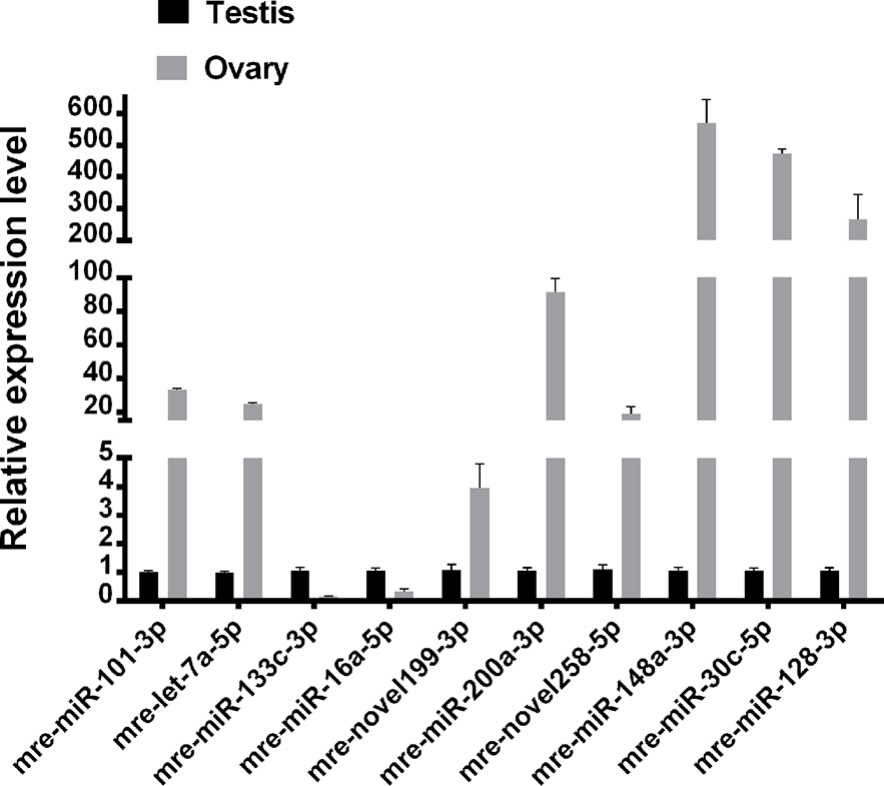
Validation of expressed miRNAs using qRT-PCR.

Ten representative differentially expressed miRNAs were assessed by qRT-PCR in this study, comprising two novel miRNAs (mre-novel258-5p, mre-novel199-3p) and eight other miRNAs (mre-miR-101-3p, mre-let-7a-5p, mre-miR-133c-3p, mre-miR-16a-5p, mre-miR-148-3p, mre-miR-200a-3p, and mre-miR-30c-5p) with roles in the regulation of sex-related gene expression in the gonadal functional maintenance and gametogenesis signaling pathways. The expression patterns of the miRNAs measured using qRT-PCR corresponded to those obtained by high-throughput sequencing, thereby confirming the accuracy and reliability of the sequencing results used in the functional analyses (**Figure 5**).

## Discussion

### miRNAs Identified in *M. reevesii* During the Sexual Maturity Period

We identified 25 conserved and 379 novel miRNA sequences, which were classified into 23 known miRNA families in *M. reevesii*. The novel miRNA sequences were mainly 21 nt (19.5%) or 22 nt (41.7%) in length, with a similar proportion compared to the conserved miRNAs. We also observed a 5′ uridine nucleotide bias in 51.5% of the novel miRNA sequences.

The most abundant miRNAs were mre-let-7a-5p, mre-let-7e-5p, mre-miR-101-3p, mre-miR-148a-3p, mre-miR-16a-5p, mre-miR-128-3p, mre-miR-1b-3p, mre-miR-200b-3p, mre-miR-30c-5p, and mre-miR-200a-3p, where each had several thousand reads. In addition, 44 novel miRNAs were identified with differential expression in the testis and ovary.

### Common Signaling Pathways for DEGs Determined by RNA-seq and Target Genes of Differentially Expressed miRNAs

**Figure 6:**
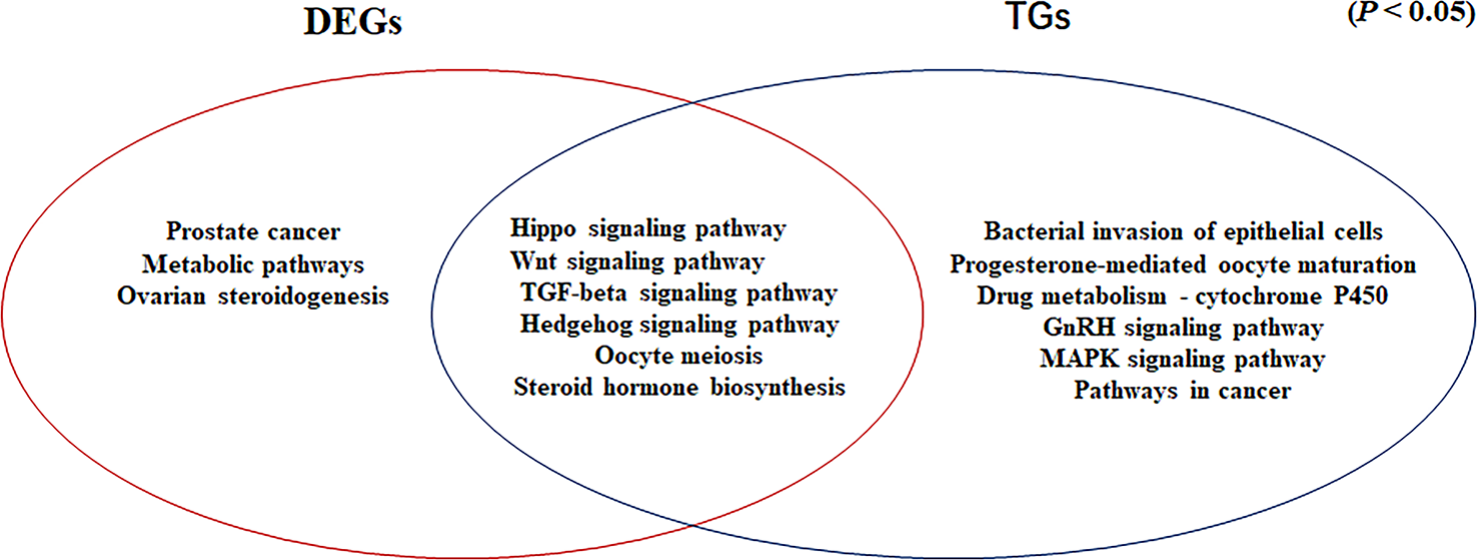
Venn diagram based on the significantly enriched pathways for the differentially expressed genes determined by RNA-seq and target genes of the differentially expressed miRNAs (*P* <0.05).

In our previous study, we showed that the Hippo, Wnt, TGF-beta, and Hedgehog signaling pathways are involved in gonadal maintenance and hormonal regulation in the adult gonad (Xiong et al., 2019). In the present study, we compared the common signaling pathways for the DEGs determined by RNA-seq and the target genes of differentially expressed miRNAs (**Figure 6**). In addition to the four signaling pathways, the Oocyte meiosis and Steroid hormone biosynthesis pathways were enriched in this study. The Oocyte meiosis signaling pathway has critical roles in controlling cell proliferation, self-renewal, differentiation, and apoptosis in most tissues and organs in diverse species (O’Hayre et al., 2014), and it could regulate the proliferation and differentiation of ovaries (Schmitt and Nebreda, 2002). The steroid hormone biosynthesis pathway is controlled by the activity of aromatase (*CYP19A1*) and interference with steroid biosynthesis might lead to impaired reproduction and alterations in sexual differentiation, growth, and development (Sanderson, 2006). Moreover, MAPK9 is a key molecule in this signaling pathway and it appears to be evolutionarily conserved for the control of oocyte growth and meiotic maturation across species (Arur, 2017). Together, these results revealed that the above six pathways play critical roles in turtle gonadal functional maintenance.

### Identification of miRNAs Involved in Testis and Ovary Functional Maintenance During the Sexual Maturity Period

**Figure 7:**
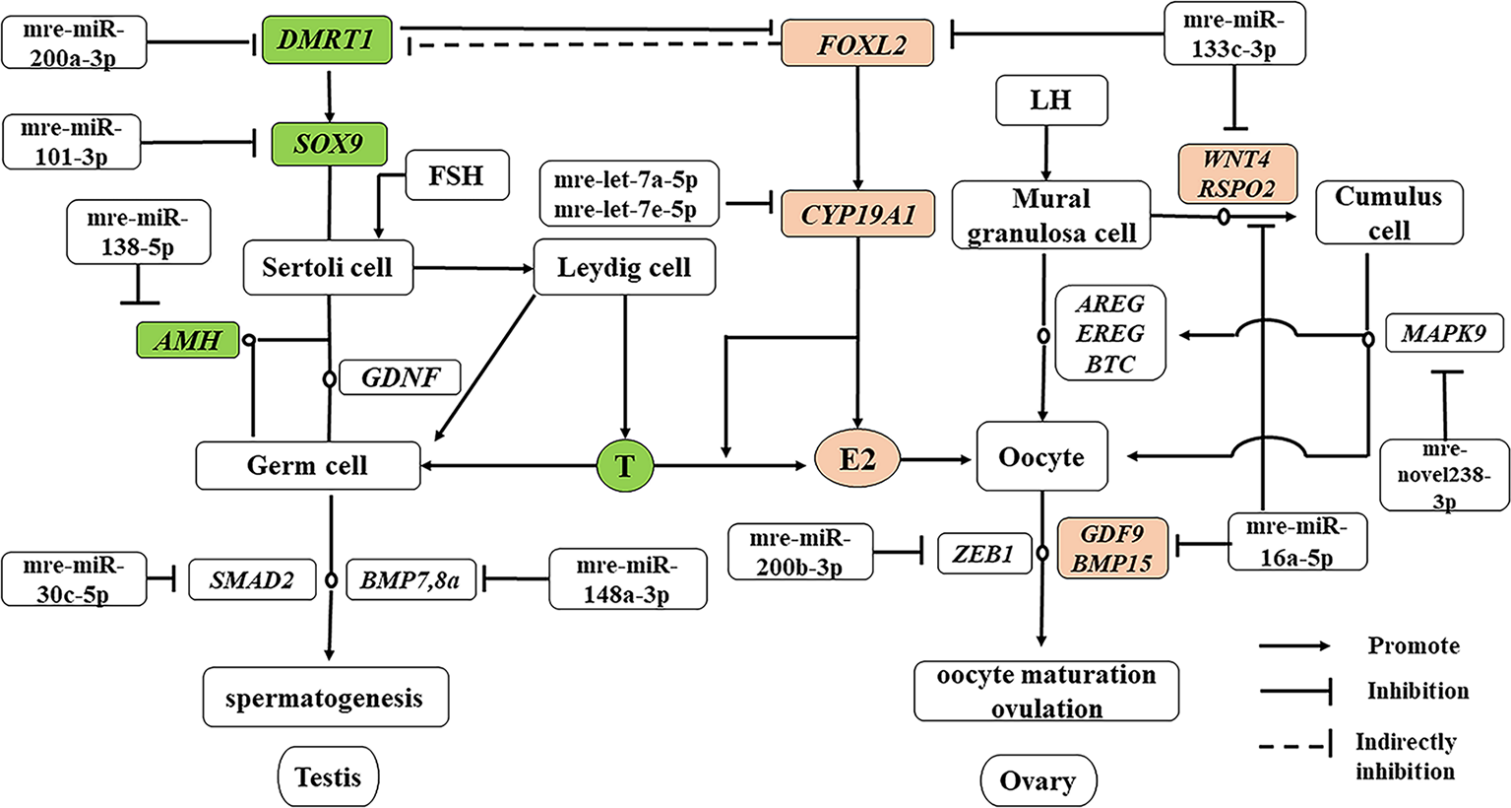
miRNA–gene network related testis and ovary functional maintenance and regulation during the sexual maturity period. The miRNAs potentially regulate the expression of their corresponding genes. Green boxed nodes represent upregulated genes in male gonads and pink boxed nodes represent upregulated genes in female gonads. T: testosterone; E2: estradiol.

In addition to producing steroid hormones, the main functions of the testes and ovaries are to produce mature gametes (Mäkelä et al., 2018). Spermatogenesis depends on testicular somatic cells (Sertoli cells and Leydig cells). Sertoli cells guide germ cells toward their spermatogenic destiny and Leydig cells produce androgens, and both of them are related to the development of spermatogenic cells during spermatogenesis (Itman et al., 2006). Luteinizing hormone increases the proliferation of Sertoli cells (Mäkelä et al., 2018). In our previous study based on RNA-seq, we showed that the antagonistic regulation of steroid hormones will maintain an appropriate balance between males and females in *M. reevesii*. The expression of key genes is involved in steroid hormone biosynthesis, such as *CYP19A1*, which is a gene encoding an enzyme that catalyzes conversion from androgens to estrogens (Matsumoto et al., 2013). Two miRNAs (mre-let-7a-5p and mre-let-7e-5p) were predicted to regulate the expression of *CYP19A1*, and the expression levels of two miRNAs were downregulated in females by more than four times. Some important sex-related genes are expressed in testis, such as doublesex and mab-3 related transcription factor 1 (*DMRT1*), anti-Müllerian hormone (*AMH*), SRY-box transcription factor 9 (*SOX9*), bone morphogenetic protein 7 (*BMP7*), bone morphogenetic protein 8a (*BMP8a*), and SMAD family member 2 (*SMAD2*), which are necessary to maintain spermatogenesis (Mäkelä et al., 2018). *DMRT1* is a candidate master male sex-determining gene in TSD turtles such as *T. scripta* (Ge et al., 2017). Although Dmrt1 is not required for male sex determination in the mouse, it is essential for maintaining the Sertoli cell phenotype in postnatal mammalian testes (Matson et al., 2011). *DMRT1* was predicted to be the target of mre-miR-200a-3p and the expression of mre-miR-200a-3p was downregulated in males. *SOX9* is essential for testicular organogenesis (Mäkelä et al., 2018). Mre-miR-101-3p was predicted to target *SOX9* and the expression of mre-miR-101-3p was downregulated in males (Yan et al., 2018). In mammals, *AMH* is not expressed in the adult testes (Xu et al., 2019), but in the adult ovary, and it is necessary for granulosa cell differentiation (Gruijters et al., 2003). But in the TSD turtle, *AMH* was upregulated in testes and downregulated in ovaries. Mre-miR-138-5p was predicted to target *AMH* and the expression of mre-miR-138-5p was upregulated in females. Mre-miR-30c-5p was predicted to target *SMAD2* and mre-miR-148a-3p was predicted to target *BMP8a*. In the adult ovary, oocytes are surrounded by somatic cell-derived granulosa cells and cumulus cells, which control oocyte maturation (Fan et al., 2009). Ovulation is initiated by a surge in luteinizing hormone, and forkhead box L2 (*FOXL2*), Wnt family member 4 (*WNT4*), R-spondin 2 (*RSPO2*), amphiregulin (*AREG*), epiregulin (*EREG*), betacellulin (*BTC*), and mitogen-activated protein kinase 9 (*MAPK9*) induce a gene activation network in the mural granulosa and cumulus cells to co-ordinate processes including oocyte maturation and ovulation (Park et al., 2004), which require oocyte participation through the actions of oocyte-secreted factor 15 (*BMP15*) and growth differentiation factor 9 (*GDF9*) (Otsuka et al., 2011). Thus, *GDF9* and *BMP15* may act in synergy to promote oocyte maturation (Richani and Gilchrist, 2017). In this regulatory network, mre-miR-133c-3p targeted both *WNT4* and *FOXL2*, whereas mre-miR-16a-5p targeted both *GDF9* and *RSPO2*, and mre-novel238-3p targeted *MAPK9*. mre-miR-200b-3p was strongly expressed in the adult ovary, where it suppressed the expression of the transcriptional repressor zinc finger E-box binding homeobox 1 (*ZEB1*) (Hasuwa et al., 2013) to maintain normal ovulation in the turtle (**Figure 7**).

Male and female TSD turtles have the same genetic background, and their gonads have functional sex reversal potential. However, adult TSD turtles do not undergo sex reversal spontaneously. Our results showed that miRNAs were involved in the antagonistic regulation of steroid hormones which would maintain an appropriate balance between males and females.

## Conclusions

The present study is the first to examine the miRNA expression profiles in the testis and ovary of *M. reevesii* during the sexual maturity period. We identified 60 miRNAs with differential expression in the testis and ovary. The 221 predicted target genes of these differentially expressed miRNAs also exhibited sex-biased expression in the adult gonads of *M. reevesii* according to transcriptomic analysis. We identified several miRNA/target gene pairs, i.e., mre-let-7a-5p/mre-let-7e-5p and *CYP19A1*, mre-miR-200a-3p and *DMRT1*, mre-miR-101-3p and *SOX9*, and mre-miR-138-5p and *AMH*, which include genes that encode key enzymes and molecules related to sexual functional maintenance and steroidogenic pathways. These results suggest the existence of a complex regulatory network after sex differentiation in the turtle and the potential importance of miRNAs for regulating the functional maintenance of the testis and ovary during the sexual maturity period.

## Data Availability Statement

The datasets generated for this study can be found in the Sequencing data for miRNA have been deposited to Sequence Read Archive at the National Center for Biotechnical Information under accession number PRJNA542219.

## Ethics Statement

Procedures involving animals and their care were approved by the Animal Care and Use Committee of Anhui Normal University.

## Author Contributions

LX and LN conceived the idea and designed the study. MY, KZ, ZW performed sample collection and RNA extractions. LX and JL analyzed the data. SG, NS, and JT performed qRT-PCR analyses. LX and LN prepared the manuscript. All authors reviewed and approved the final manuscript.

## Funding

This research was supported by the National Natural Science Foundation of China (NSFC, No. 31970499), the Research Fund of the Key Laboratory of Biotic Environment and Ecological Safety of Anhui province, and the Natural Science Research Project in Colleges and Universities of Anhui province (No. KJ2017A254). The funders had no role in study design, data collection and analysis, decision to publish, or preparation of the manuscript.

## Conflict of Interest

The authors declare that the research was conducted in the absence of any commercial or financial relationships that could be construed as a potential conflict of interest.

## Supplementary Material

Supplementary Figure 1
*M. reevesii* ovary and testis tissues. (A) Testis; (B) Ovary. Blue bar = 1,000 µm; Digital microscopic system VHX-5000.

Supplementary Figure 2 Length distribution of six small RNA libraries.

Supplementary Table 1 The primers of qRT-PCR validation.

Supplementary Table 2 Data filtering results.

Supplementary Table 3 The conserved miRNAs in *M. reevesii*.

Supplementary Table 4 The novel miRNAs in *M. reevesii*.

Supplementary Table 5 The list of differentially expressed miRNAs.

Supplementary Table 6 The target genes predicted for the differentially expressed miRNAs.

Supplementary Table 7 The significant GO terms.

Supplementary Table 8 The significant KEGG pathways.
