# Supplementary figures and images for: Comparison of Adult Testis and Ovary MicroRNA Expression Profiles in Reeves’ Pond Turtles (Mauremys reevesii) With Temperature-Dependent Sex Determination
Source: Front Genet. 2020 Mar 2;11:133. doi: 10.3389/fgene.2020.00133 (PMC7061903; doi:10.3389/fgene.2020.00133)

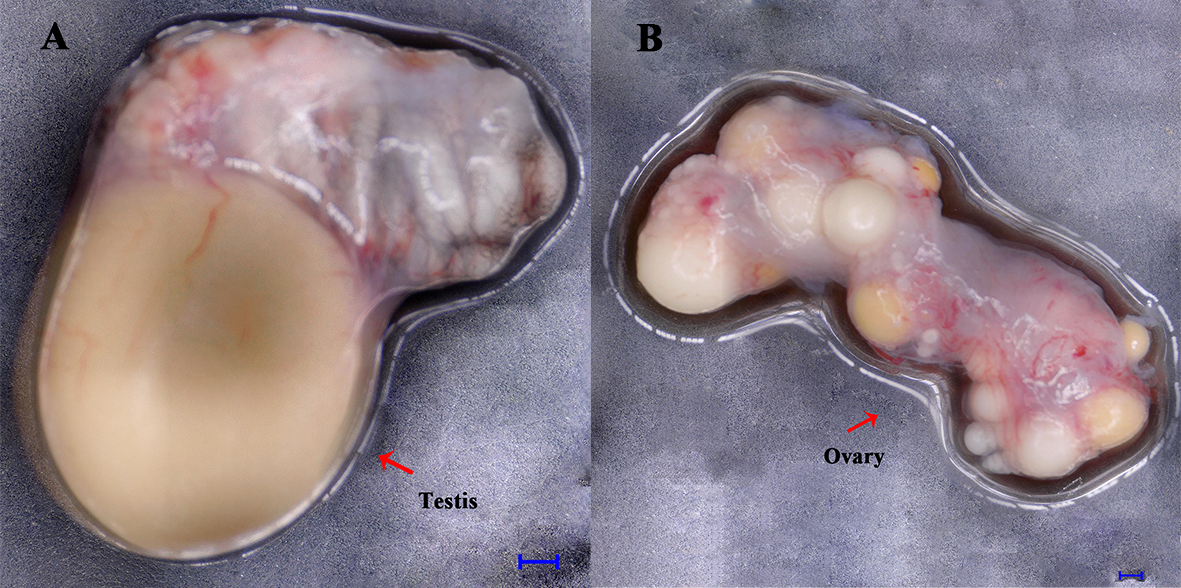

Supplement: Supplementary Figure 1 — M. reevesii ovary and testis tissues. (A) Testis; (B) Ovary. Blue bar = 1,000 µm; Digital microscopic system VHX-5000. [file Image_1.tif]

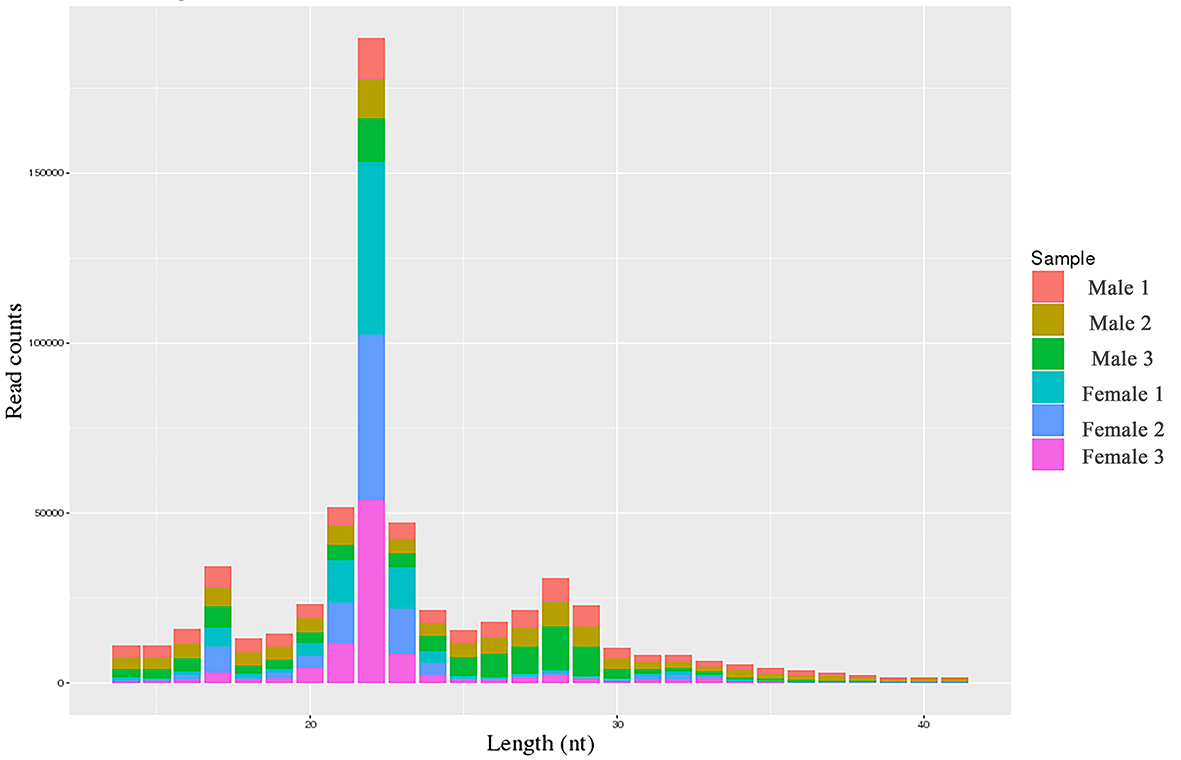

Supplement: Supplementary Figure 2 — Length distribution of six small RNA libraries. [file Image_2.tif]
